# App‐based pelvic floor muscle training in pregnant and postnatal women: A prospective cohort study exploring factors associated with prevention and improvement of urinary incontinence

**DOI:** 10.1002/hsr2.781

**Published:** 2022-08-18

**Authors:** Erika Löjdahl, Anna Lindam, Ina Asklund

**Affiliations:** ^1^ Department of Public Health and Clinical Medicine Umeå University Umeå Sweden; ^2^ Department of Public Health and Clinical Medicine, Unit of Research, Education and Development, Östersund Hospital Umeå University Umeå Sweden

**Keywords:** mobile applications, pelvic floor muscle training, postpartum period, pregnant women, self‐management, urinary incontinence

## Abstract

**Background and Aims:**

Pelvic floor muscle training (PFMT) is recommended for continent pregnant women and postnatal women experiencing urinary incontinence (UI). The app Tät® has been developed for the treatment of stress UI with a focus on PFMT. The aim of this study was to investigate factors associated with the improvement of incontinence symptoms and retained continence in pregnant and postnatal women who used the app.

**Methods:**

A prospective cohort study was carried out based on user questionnaires from the app Tät®. We included pregnant and postnatal women who answered the inclusion questionnaire between June 19, 2019 and September 19, 2020. The questionnaire included questions about the frequency and amount of leakage, the impact that UI has on everyday life, and experienced improvements at follow‐up. We analyzed factors associated with improvement and retained continence using logistic regression.

**Results:**

We included 10,307 pregnant and 13,670 postnatal women, and 44% of the pregnant women and 52% of the postnatal women were incontinent. A total of 3680 women were included in the follow‐up analysis, and 52% of the pregnant incontinent women and 73% of the postnatal incontinent women experienced improvement. Pregnant women who performed PFMT and used the app at least once per week had increased odds of improvement (odds ratio [OR]: 1.83, 95% confidence interval [CI]: 1.01–3.29 and OR: 3.38, 95% CI: 1.94–5.90, respectively) compared to those who performed no training and had no app usage. Postnatal women who used the app at least once per week and had more severe incontinence had increased odds of improvement (OR: 4.26, 95% CI: 2.37–7.64 and OR: 1.11, 95% CI: 1.05–1.16, respectively).

**Conclusions:**

The app Tät® is widely used by pregnant and postnatal women in Sweden for the prevention and treatment of UI. Majority of the women with incontinence experienced improvement after using the app. Regular PFMT and app use seemed to be important factors for experiencing improvement.

## 1 INTRODUCTION

Urinary incontinence (UI), defined as involuntary leakage of urine,^1^ is common and affects 25%–45% of all women.^2^ Stress urinary incontinence (SUI) is leakage during physical exercise, coughing, or sneezing, and is the most frequent subtype.^1^ Pregnancy and childbirth are risk factors for developing UI, especially SUI.^2^ During pregnancy, the prevalence of SUI ranges from 18.6% to 60% and it increases during late pregnancy.^3^ During the 3 months after childbirth, the prevalence of UI is around 30%,^4^ and the frequency of postpartum SUI is higher in women who have a vaginal delivery than those who have a cesarean Section.^5^

The present‐day recommendations are that pelvic floor muscle training (PFMT) should be offered to all continent pregnant women and postnatal women experiencing UI.^6^ However, there is insufficient evidence that PFMT is effective for treating UI during pregnancy and postpartum and there is a need for further investigation into PFMT in these specific groups.^7^, ^8^

UI can have a significant impact on quality of life.^9^ Despite this, only 31% of those who suffer seek professional help, and one reason could be the feeling of embarrassment when talking to a physician about it.^10^, ^11^ PFMT is the first‐line treatment for all types of UI,^2^ and a mobile app is an easily available and cost‐effective way to offer PFMT.^12^ Via an app, women who are not willing to ask for face‐to‐face care can receive the instructions through a smartphone. The app Tät® has been developed for the self‐treatment of SUI, with a focus on PFMT. Its effects have been evaluated in a randomized‐controlled trial (RCT), which showed significant improvements in terms of clinical symptoms, number of leakages, and quality of life, compared to a control group.^13^ Pregnant women were not included in the RCT. Since 2015, the app has been free to download from App Store and Google Play and we follow its use in the real world.^14^

The aim of this study was to investigate the use of the freely available app Tät® during pregnancy and the postnatal period. Specifically, the aim was to investigate factors associated with the improvement of incontinence symptoms in pregnant and postnatal women who used the app Tät®. It also investigated factors associated with retained continence in pregnant women who used the app.

## 2 MATERIALS AND METHODS

**Figure 1:**
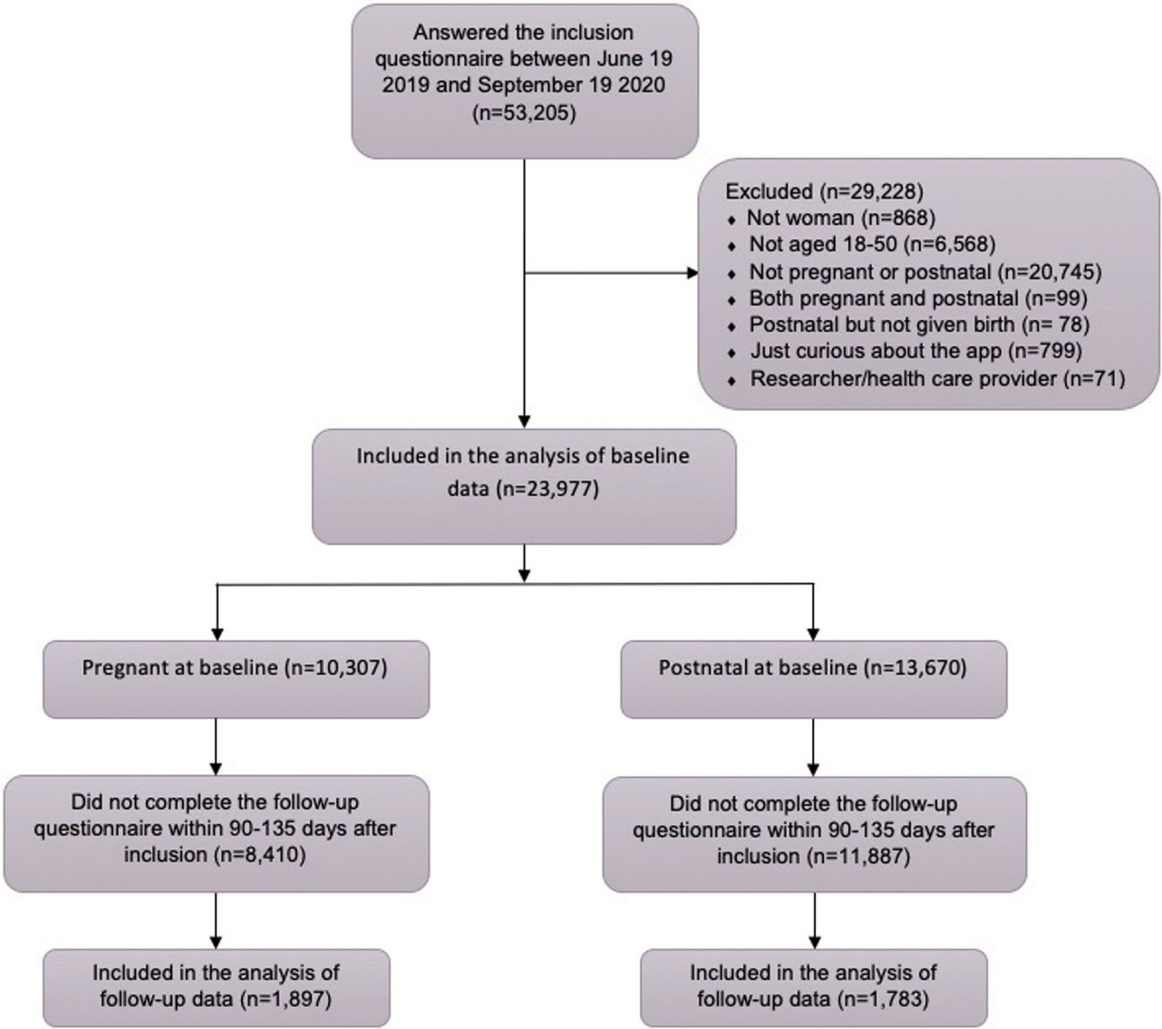
Flowchart of the study population

This was a prospective cohort study based on data from the mobile app Tät®. Inclusion criteria were as follows: users who answered the inclusion questionnaire between June 19, 2019 and September 19, 2020; stated that they were female; were 18–50 years old; and were either pregnant or had given birth no more than 3 months before downloading the app (i.e., were postnatal). Follow‐up questionnaires completed within 90–135 days after inclusion were included since we only wanted answers from active users (Figure 1).

The app Tät® was developed within the eContinence project by Eva Samuelsson, Malin Sjöström, and Göran Umefjord in collaboration with software engineers at Information and Communications Technology Services and System Development, Umeå University. Since 2015, the app has been free to download from App Store and Google Play and is currently available in eight languages (English, Swedish, Finnish, Danish, Norwegian, Spanish, German, and Arabic). Tät® consists of a self‐management program for SUI based on PFMT, which includes 12 exercises that increase in intensity and difficulty. The app also contains lifestyle advice, as well as information about SUI, the pelvic floor, and factors related to incontinence. Tät® is CE‐marked as a medical device class 1 (LVFS 2003:11).

When the users downloaded the app, an inclusion questionnaire appeared containing questions about age, gender, country, place of residence, education, the reason for downloading, pregnancy, stage of pregnancy, childbirth during the last 3 months, the number of childbirths, mode of delivery, and incontinence symptoms. The questionnaires included the International Consultation on Incontinence Questionnaire‐Urinary Incontinence Short Form (ICIQ‐UI SF), which consists of questions about the frequency and amount of leakage as well as the overall impact that UI has on everyday life.^15^ The responses make up a total score of between 0 and 21, which can be divided into different severity categories: 1–5 (slight), 6–12 (moderate), 13–18 (severe), and 19–21 (very severe).^16^ We defined users who answered that they had both some amount of leakage and some frequency of leakage as incontinent.

The ICIQ‐UI SF also includes a self‐diagnostic question about the situations in which leakage occurs. Users who answered that they had leakage when they coughed, sneezed, or were physically active but had no leakage before they reached the toilet were defined as having stress UI. Users who answered that they had leakage before they reached the toilet but not when they coughed, sneezed, or were physically active were defined as having urgency UI. Users who answered that they had both symptoms of stress UI and urgency UI were defined as having mixed UI.

After 3 months, a follow‐up questionnaire appeared with questions about pregnancy, childbirth during the last 3 months, use of the app, frequency of performed PFMT, and incontinence symptoms based on the ICIQ‐UI SF. The question about PFMT frequency had five response options: never, less than once a week, 1–6 times per week, daily, three times daily, or more. The question about app use also had five response options: not at all, about once a month, about once a week, about once a day, and several times a day. There was also a question about the subjective change in symptoms based on the validated patient's global impression of improvement (PGI‐I), which has seven response options ranging from “very much worse” to “very much better.”^17^ We defined incontinent users who answered that their condition was “a little better,” “much better,” and “very much better” on the PGI‐I as showing “improvement.”

Answering the questionnaires was optional and the app could be fully used even if the users declined to participate in the study. The users were informed that their responses were sent anonymously and encrypted to the research database. The users gave their informed consent by ticking a box in the questionnaires. A unique app identification linked the user's response at inclusion to the same user's response at follow‐up, but the answers could not be traced back to a specific person. No information about the user's name, social security number, email address, IP address, or phone number was ever collected.

### 2.1 Statistical analyses

Descriptive statistics were presented as frequencies with percentages and mean values with standard deviations. Differences between groups were analyzed using the *χ* ^2^ test for categorical variables and analysis of variance (ANOVA) for continuous variables. Changes in mean ICIQ‐UI SF scores were analyzed using a paired samples *t*‐test, and differences in mean change between groups were analyzed using ANOVA.

Potential confounders were included in the analysis of association: age, education level, stage of pregnancy, mode of delivery, childbirth during the last 3 months, number of childbirths, ICIQ‐UI SF score at inclusion, type of incontinence, PFMT frequency, and app usage frequency. If a variable like PFMT and app use frequency had too few answers in one response category to perform analysis, that response category collapsed with the closest category. All variables were first analyzed in separate logistic regression models, and the associations with “improvement” or “retained continence” were estimated. Variables that had a *p* < 0.2 were included in the multiple logistic regression models. Odds ratios (OR) and 95% confidence intervals (CIs) were estimated. Age and ICIQ‐UI SF scores were analyzed as continuous variables, whereas the other variables were analyzed as categorical variables. *p* < 0.05 were considered statically significant in all analyses.

All data were analyzed using SPSS® version 27 and there were no missing values in the analyses.

## 3 RESULTS

A total of 53,205 app users answered the inclusion questionnaire. Of these 23,977 met the inclusion criteria for baseline analysis, of which 10,307 (43%) were pregnant and 13,670 (57%) were postnatal (Figure 1).

In pregnant women, the mean age was 31 years. The majority lived in Sweden (*n* = 8962) and had a university education (*n* = 7625). Two‐thirds (*n* = 6878) had not experienced childbirth when downloading the app, and most women (*n* = 8385) had downloaded it for preventive purposes. The proportion of incontinent women was higher in Weeks 27+ of pregnancy than in Weeks 0–13 of pregnancy (51.1% vs. 33.8%). Around two‐thirds (*n* = 3131) of the incontinent women had SUI (Table 1).

**Table 1:** Baseline characteristics of pregnant women who answered the inclusion questionnaire

Baseline characteristics	Pregnant women (*n* = 10,307)			Differences between the groups, *p*‐value^a^
	Pregnancy week 0–13 (*n* = 2218)	Pregnancy week 14–26 (*n* = 4513)	Pregnancy week 27+ (*n* = 3576)	
Age, mean (SD)	31.00 (4.279)	30.57 (4.178)	30.80 (4.303)	<0.001
*Country, n (%)*				<0.001
Sweden	1880 (84.8)	3931 (87.1)	3151 (88.1)	
Norway, Denmark, Finland, Iceland	173 (7.8)	351 (7.8)	221 (6.2)	
Other	165 (7.4)	231 (5.1)	204 (5.7)	
*Language, n (%)*				<0.001
Swedish	1861 (83.9)	3,924 (86.9)	3,124 (87.4)	
English	114 (5.1)	227 (5.0)	189 (5.3)	
Other	243 (11.0)	362 (8.0)	263 (7.4)	
*Place of residence, n (%)*				0.085
Rural area	335 (15.1)	669 (14.8)	570 (15.9)	
The town with <50,000 inhabitants	411 (18.5)	928 (20.6)	739 (20.7)	
The town with 50,000 to 1 million inhabitants	840 (37.9)	1749 (38.8)	1353 (37.8)	
The city with >1 million inhabitants	632 (28.5)	1167 (25.9)	914 (25.6)	
*Highest level of education, n (%)*				0.006
6 years of school or less	23 (1.0)	47 (1.0)	40 (1.1)	
7–9 years of school	25 (1.1)	64 (1.4)	53 (1.5)	
10–12 years of school	457 (20.6)	1076 (23.8)	897 (25.1)	
University	1713 (77.2)	3326 (73.7)	2586 (72.3)	
*Reason for downloading the app, n (%)*				0.058
Treatment	378 (17.0)	845 (18.7)	699 (19.5)	
Prevention	1840 (83.0)	3668 (81.3)	2877 (80.5)	
*Number of childbirths, n (%)*				<0.001
None	1362 (61.4)	3108 (68.9)	2408 (67.3)	
One	602 (27.1)	1091 (24.2)	864 (24.2)	
Two	204 (9.2)	259 (5.7)	233 (6.5)	
Three or more	50 (2.3)	55 (1.2)	71 (2.0)	
Incontinent, *n* (%)	750 (33.8)	1929 (42.7)	1829 (51.1)	<0.001
*Type of incontinence, n (%)* b				<0.001
Stress urinary incontinence^b^	477 (63.6)	1353 (70.1)	1301 (71.1)	
Urgency urinary incontinence^b^	75 (10.0)	110 (5.7)	92 (5.0)	
Mixed urinary incontinence^b^	152 (20.3)	323 (16.7)	288 (15.7)	
Other^b^	46 (6.1)	143 (7.4)	148 (8.1)	
ICIQ‐UI SF score, mean (SD)^b^	6.39 (3.219)	6.20 (3.180)	6.49 (3.333)	0.022
*Symptom severity, n (%)* b				0.197
Slight^b^	391 (52.1)	1,050 (54.4)	933 (51.0)	
Moderate^b^	314 (41.9)	764 (39.6)	764 (41.8)	
Severe/very severe^b^	45 (6.0)	115 (6.0)	132 (7.2)	

Abbreviations: ANOVA, analysis of variance; ICIQ‐UI SF, International Consultation on Incontinence Modular Questionnaire‐Urinary Incontinence Short Form.

^a^ *p*‐values were calculated using the *χ* ^2^ test for categorical variables and ANOVA for continuous variables.

^b^ In those with incontinence.

In postnatal women, the mean age was 32 years and the majority had only had a vaginal delivery (*n* = 11,846). Most women lived in Sweden (*n* = 12,371) and had university education (*n* = 9499). The proportion of incontinent women was higher in those who had only had a vaginal delivery compared to those who had only had a cesarean section (53.1% vs. 33.4%). Almost half of the incontinent women had SUI (*n* = 3524) (Table 2).

**Table 2:** Baseline characteristics of postnatal women who answered the inclusion questionnaire.

Baseline characteristics	Postnatal women (*n* = 13,670)			Differences between the groups, *p*‐value^a^
	Vaginal delivery (*n* = 11,846)	Cesarean section (*n* = 874)	Both delivery modes (*n* = 950)	
Age, mean (SD)	31.13 (4.357)	32.32 (4.653)	33.18 (4.277)	<0.001
*Country, n (%)*				0.010
Sweden	10,756 (90.8)	770 (88.1)	845 (88.9)	
Norway, Denmark, Finland, Iceland	492 (4.2)	39 (4.5)	51 (5.4)	
Other	598 (5.0)	65 (7.4)	54 (5.7)	
*Language, n (%)*				0.025
Swedish	10,566 (89.2)	754 (86.3)	835 (87.9)	
English	536 (4.5)	49 (5.6)	39 (4.1)	
Other	744 (6.3)	71 (8.1)	76 (8.0)	
*Place of residence, n (%)*				<0.001
Rural area	2079 (17.6)	131 (15.0)	192 (20.2)	
The town with <50,000 inhabitants	2735 (23.1)	171 (19.6)	256 (26.9)	
The town with 50,000 to 1 million inhabitants	4191 (35.4)	299 (34.2)	279 (29.4)	
The city with >1 million inhabitants	2841 (24.0)	273 (31.2)	223 (23.5)	
*Highest level of education, n (%)*				0.124
6 years of school or less	133 (1.1)	11 (1.3)	19 (2.0)	
7–9 years of school	211 (1.8)	19 (2.2)	24 (2.5)	
10–12 years of school	3271 (27.6)	227 (26.0)	256 (26.9)	
University	8231 (69.5)	617 (70.6)	651 (68.5)	
*Reason for downloading the app, n (%)*				<0.001
Treatment	4858 (41.0)	226 (25.9)	423 (44.5)	
Prevention	6988 (59.0)	648 (74.1)	527 (55.5)	
*Number of childbirths, n (%)*				0.000
One	6825 (57.6)	633 (72.4)	52 (5.5)	
Two	3672 (31.0)	201 (23.0)	627 (66.6)	
Three or more	1349 (11.4)	40 (4.6)	271 (28.5)	
Incontinent, *n* (%)	6292 (53.1)	292 (33.4)	555 (58.4)	<0.001
*Type of incontinence, n (%)* b				<0.001
Stress urinary incontinence^b^	3111 (49.4)	145 (49.7)	268 (48.3)	
Urgency urinary incontinence^b^	929 (14.8)	49 (16.8)	76 (13.7)	
Mixed urinary incontinence^b^	1684 (26.8)	47 (16.1)	151 (27.2)	
Other^b^	568 (9.0)	51 (17.5)	60 (10.8)	
ICIQ‐UI SF score, mean (SD)^b^	7.24 (3.783)	6.36 (3.467)	8.13 (4.160)	<0.001
*Symptom severity, n (%)* b				<0.001
Slight^b^	2681 (42.6)	164 (56.2)	187 (33.7)	
Moderate^b^	2890 (45.9)	106 (36.3)	266 (47.9)	
Severe/very severe^b^	721 (11.5)	22 (7.5)	102 (18.4)	

Abbreviations: ANOVA, analysis of variance; ICIQ‐UI SF, International Consultation on Incontinence Modular Questionnaire‐Urinary Incontinence Short Form.

^a^ *p*‐values were calculated using the *χ* ^2^ test for categorical variables and ANOVA for continuous variables.

^b^ In those with incontinence.

A total of 3680 women were included in the follow‐up analysis. More than two‐thirds of all women had used the app (72.9%) and performed PFMT (69.5%) at least once per week during the last month. A higher proportion of women who were in Weeks 14–26 and Weeks 27+ of pregnancy than those in Weeks 0–13 of pregnancy had used the app and performed PFMT daily (23.6% and 29.2% vs. 16.2%). In postnatal women, the frequency of PFMT training and app usage was similar regardless of the mode of delivery (Tables 3 and 4).

**Table 3:** Follow‐up data of PFMT and app usage in pregnant women who answered the follow‐up questionnaire, according to groups assigned to inclusion

	Pregnant women (*n* = 1897)			Differences between the groups, *p*‐value^a^
	Pregnancy week 0–13 (*n* = 401)	Pregnancy week 14–26 (*n* = 804)	Pregnancy week 27+ (*n* = 692)	
*Frequency of performed PFMT in the past 4 weeks, n (%)*				<0.001
Never	48 (12.0)	88 (10.9)	63 (9.1)	
<Once/week	111 (27.7)	176 (21.9)	137 (19.8)	
1–6 times/week	177 (44.1)	350 (43.5)	290 (41.9)	
Daily	65 (16.2)	190 (23.6)	202 (29.2)	
*Use of the app since download, n (%)*				<0.001
Never	67 (16.7)	88 (10.9)	118 (17.1)	
Once/month	81 (20.2)	119 (14.8)	109 (15.8)	
Once/week	120 (29.9)	232 (28.9)	167 (24.1)	
Daily	133 (33.1)	365 (45.4)	298 (43.1)	

Abbreviation: PFMT, pelvic floor muscle training.

^a^ *p*‐values were calculated using the *χ* ^2^ test for independence.

**Table 4:** Follow‐up data of PFMT and app usage in postnatal women who answered the follow‐up questionnaire, according to groups assigned to inclusion

	Postnatal women (*n* = 1783)			Differences between the groups, *p*‐value^a^
	Vaginal delivery (*n* = 1586)	Cesarean section (*n* = 82)	Both delivery modes (*n* = 115)	
*Frequency of performed PFMT in the past 4 weeks, n (%)*				0.309
Never	107 (6.7)	11 (13.4)	9 (7.8)	
<Once/week	328 (20.7)	19 (23.2)	24 (20.9)	
1–6 times/week	667 (42.1)	34 (41.5)	47 (40.9)	
Daily	484 (30.5)	18 (21.9)	35 (30.5)	
*Use of the app since download, n (%)*				0.083
Never	130 (8.2)	13 (15.9)	10 (8.7)	
Once/month	228 (14.4)	18 (22.0)	16 (13.9)	
Once/week	409 (25.8)	15 (18.3)	28 (24.3)	
Daily	819 (51.6)	36 (43.9)	61 (53.0)	

Abbreviation: PFMT, pelvic floor muscle training.

^a^ *p*‐values were calculated using the *χ* ^2^ test for independence.

The ICIQ UI‐SF scores at inclusion were higher in postnatal women than in pregnant women. Women who were in Weeks 0–13 of pregnancy at inclusion had no improvement in the ICIQ‐UI SF score after 3 months. Women who were in Weeks 27+ of pregnancy improved more than women who were in Weeks 14–26 of pregnancy (1.90 vs. 0.49 points). The postnatal women improved on average by 2.61 points, and there was no significant difference between women who had had different modes of delivery (*p* = 0.734) (Table 5).

**Table 5:** Change in ICIQ‐UI SF scores in pregnant and postnatal women with incontinence at inclusion who also answered the follow‐up questionnaire, according to groups assigned at inclusion

	ICIQ‐UI SF score at inclusion, mean (SD)	ICIQ‐UI SF score at follow‐up, mean (SD)	Difference, mean (95% confidence Interval)	Differences in scores within groups, *p*‐value^a^	Differences in mean change between groups, *p*‐value^b^
*Pregnant women (n = 826)*					<0.001
Pregnancy week 0–13 (*n* = 113)	5.94 (2.753)	6.07 (3.734)	0.13 (0.37–0.64)	0.602	
Pregnancy week 14–26 (*n* = 345)	5.68 (2.632)	5.19 (3.457)	0.49 (0.13–0.84)	0.007	
Pregnancy week 27+ (*n* = 368)	6.41 (3.400)	4.51 (4.298)	1.90 (1.51–2.28)	<0.001	
*Postnatal women (n = 982)*					0.734
Vaginal delivery (*n* = 888)	7.20 (3.647)	4.61 (3.746)	2.59 (2.34–2.84)	0.000	
Cesarean section (*n* = 28)	6.71 (3.857)	4.29 (4.995)	2.43 (0.53–5.32)	0.014	
Both delivery modes (*n* = 66)	8.38 (4.292)	5.42 (4.547)	2.95 (1.97–3.94)	<0.001	

Abbreviations: ANOVA, analysis of variance; ICIQ‐UI SF, International Consultation on Incontinence Modular Questionnaire‐Urinary Incontinence Short Form.

^a^ *p*‐values were calculated using paired samples *t*‐test.

^b^ *p*‐values were calculated using ANOVA.

**Figure 2:**
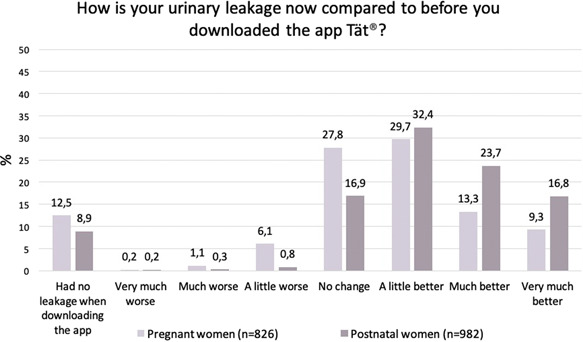
PGI‐I and definition of the outcome measure “improvement.” Pregnant and postnatal women with incontinence at inclusion who responded to the follow‐up questionnaire. PGI, patient global impression of improvement.

Of the 826 pregnant women that were incontinent at inclusion, 432 (52.3%) had improved at follow‐up (Figure 2). In the analyses of potential factors associated with “improvement,” age, education level, stage of pregnancy, number of childbirths, mode of delivery, frequency of PFMT, and frequency of app users had a *p* < 0.2 (data not shown) and were thus included in the multiple regression model. In the multiple regression model, PFMT frequency and app usage frequency were significantly associated with “improvement” (Table 6).

**Table 6:** Factors associated with “improvement” among pregnant and postnatal women with incontinence at inclusion who also answered the follow‐up questionnaire

Significant factors in the multiple logistic regression models^a^	Unadjusted OR (95% confidence interval)	*p*‐Value^b^	Adjusted OR (95% confidence interval)	*p*‐Value^b^
*Pregnant women (n = 826)*				
Frequency of performed PFMT in the past 4 weeks				
Never	1.00	Reference	1.00	Reference
<Once/week	1.46 (0.82–2.59)	0.197	1.20 (0.65–2.21)	0.570
1–6 times/week	3.61 (2.12–6.15)	<0.001	1.83 (1.01–3.29)	0.045
Daily	5.65 (3.18–10.04)	<0.001	2.37 (1.24–4.56)	0.009
Use of the app since the download				
Never	1.00	Reference	1.00	Reference
Once/month	0.95 (0.54–1.68)	0.861	1.00 (0.55–1.81)	0.988
1–6 times/week	3.86 (2.31–6.47)	<0.001	3.38 (1.94–5.90)	<0.001
Daily	5.36 (3.27–8.78)	<0.001	4.01 (2.30–6.97)	<0.001
*Postnatal women (n = 982)*				
Mode of delivery				
Vaginal delivery	1.00	Reference	1.00	Reference
Cesarean section	0.36 (0.17–0.78)	0.009	0.39 (0.17–0.90)	0.026
Both	1.24 (0.68–2.24)	0.482	1.26 (0.66–2.38)	0.486
ICIQ‐UI SF score at inclusion	1.12 (1.07–1.17)	<0.001	1.11 (1.05–1.16)	<0.001
Use of the app since the download				
Never	1.00	Reference	1.00	Reference
Once/month	1.50 (0.85–2.64)	0.166	1.50 (0.82–2.75)	0.185
1–6 times/week	4.22 (2.46–7.23)	<0.001	4.26 (2.37–7.64)	<0.001
Daily	6.88 (4.13–11.45)	<0.001	5.14 (2.86–9.22)	<0.001

Abbreviations: ICIQ‐UI SF, International Consultation on Incontinence Modular Questionnaire‐Urinary Incontinence Short Form; OR, odds ratio; PFMT, pelvic floor muscle training.

^a^ *p* < 0.2 in separate logistic regression models were included in the multiple logistic regression models.

^b^ *p*‐values were calculated using binary logistic regression.

Of the 982 postnatal women with incontinence at inclusion, 716 (72.9%) had improved at follow‐up (Figure 2). In the analyses of potential factors associated with “improvement,” the ICIQ‐UI SF score at inclusion, mode of delivery, type of incontinence, frequency of PFMT, and frequency of app users had a *p* < 0.2 (data not shown) and were thus included in the multiple regression model. In the multiple regression model, the ICIQ‐UI SF score, mode of delivery, and app usage frequency were significantly associated with “improvement” (Table 6).

Of the 1071 pregnant women that were continent at inclusion, 773 (72.2%) were still continent at follow‐up. In the analyses of potential factors associated with “retained continence,” age, PFMT frequency, and *given birth at follow‐up* had a *p* < 0.2 and were thus included in the multiple regression model (data not shown). Only age had a significant association with “retained continence” (OR: 0.95, 95% CI: 0.92–0.99, *p* = 0.007).

## 4 DISCUSSION

We found that 52% of the pregnant incontinent women and 73% of the postnatal incontinent women experienced improvement after 3 months of using the app Tät®. At least weekly PFMT and use of the app increased the odds of experiencing improvement in pregnant women. Higher symptom severity and at least weekly app usage increased the odds of experiencing improvement in postnatal women, whereas having had a cesarean section decreased the odds of experiencing improvement. Further, we found that 72% of the continent's pregnant women retained continence after 3 months of using the app, with an older age decreasing the odds of staying continent during pregnancy.

The strengths of this study include its prospective study design and a large number of participants included, which was possible due to the short, anonymous questionnaire. A further strength is the medical relevance of app‐based treatment, which can reach many women, including those not wanting to ask for face‐to‐face care. Also, the fact that we used the validated ICIQ‐UI SF and PGI‐I increases the reliability and enables comparison to other studies. Additionally, since the study was performed in the same clinical setting as the app is meant to be used, it increases the transferability to app users outside of the study.

The main limitation of the study is the lack of a control group, which means we cannot know whether the improvements are due to PFMT, other concomitant treatment, or a natural improvement occurring over time. The same applies to whether the continent pregnant women would have stayed continent regardless of whether they used the app or not. However, since PFMT and app usage were associated with improvement, we assume they contributed to the results. Moreover, we cannot know if only women that experienced an improvement continued to use the app. Furthermore, the women in this study were highly educated with 71% having a university education compared to 58.5% of all women aged 25–44 in Sweden in 2019.^18^ It is thus possible that these women are not comparable to all pregnant and postnatal women in need of treatment for UI. We nonetheless found no association between education level and improvement in incontinence symptoms, which is why we believe the same results could also be achieved by women with lower levels of education.

Of the users, 90% lived in Sweden. If we compare these women to the Swedish population, these women represented 15% of all pregnant/postnatal women in Sweden for the 15‐month period of the study, based on the number of childbirths in Sweden in 2019 (114,600).^19^ Of the postnatal women, 13.3% had had a cesarean section which reflects reality quite well as 17.7% of all childbirths in Sweden in 2019 were cesarean sections.^19^ Our findings thereby show that Tät® is widely used by pregnant and postnatal women in Sweden, and it reaches women who have had vaginal deliveries and those who have had a cesarean section.

Fifteen percent of those who answered the inclusion questionnaire also answered the follow‐up questionnaire. A low follow‐up rate was expected as we had no personal contact with the participants, together with the experience of an earlier study on the real‐world use of the app.^14^ One explanation for the low follow‐up rate could be that women who were continent and had downloaded the app for prevention of UI were lacking the motivation for continuing using the app. An earlier study found that incontinent users had a higher response rate than continent users, indicating that they were more likely to stick with the program.^14^ Another explanation could be that continent women and those with less difficult symptoms only downloaded the app for information and never intended to continue using it for 3 months. It is also possible that only users that experienced an improvement continued using the app which thereby could have influenced the results.

Postnatal women had both a higher level of severity of incontinence and larger improvements than pregnant women. An earlier study found that more severe incontinence led to a greater ICIQ‐UI SF score reduction,^14^ so it is, therefore, possible that a higher symptom severity increases the possibility of improvement. However, these findings might also indicate that PFMT is not as beneficial for pregnant women as for postnatal women, which can be deemed to agree with a study by Woldringh et al.^20^ which found that PFMT was not effective during pregnancy for women who were already affected by UI.

Only pregnant women who were in mid or late pregnancy at inclusion had an improved ICIQ‐UI SF score. It has previously been suggested that the result of PFMT is highly dependent on the degree and duration of the training.^21^ It is thus possible that women in early pregnancy did not improve due to a lower frequency of PFMT and app usage.

All postnatal women had an improved ICIQ‐UI SF score. It has previously been suggested that a change of 2.5 points in the ICIQ‐UI SF score is clinically relevant at group level,^22^ and therefore our results of 2.61 points' improvement among postnatal women are clinically significant. We, therefore, believe that the incontinence symptoms of all postnatal women, independent of the mode of delivery, could improve via app‐based PFMT.

The association between improvement and a higher frequency of PFMT and app use has also been found by others, which strengthens the suggestion that treatment adherence is important for improvement.^21^, ^23^ For example, Nyström et al.^24^ found that at least weekly PFMT and use of the app increased the odds of improvement among nonpregnant/nonpostnatal women. Furthermore, they found a correlation between the frequency of PFMT and the frequency of app use, suggesting that the effects of these factors might overlap. However, the app also includes information and lifestyle advice, which could explain the beneficial effects of only using the app.

The finding that a higher ICIQ‐UI SF score at inclusion would increase the odds of improvement is in contrast to what earlier studies have found.^24^, ^25^, ^26^, ^27^, ^28^ However, these studies used different outcome measurements. For example, Lindh et al.^23^ found that a higher ICIQ‐UI SF score predicted a reduction of ≥3 points, whereas a lower ICIQ‐UI SF score predicted higher treatment satisfaction.

The finding that a cesarean section would decrease the odds of improvement has not been suggested by others. Earlier studies have found no correlation between obstetric history and the effects of PFMT.^25^, ^29^ However, since most women in this study had only had vaginal deliveries, the uneven distribution might have affected our analysis of association.

Our findings show that a PFMT program within a mobile app can reach many pregnant and postnatal women. Our results do not indicate that such an app should only be offered to certain groups of pregnant and postnatal women. As treatment via a mobile app is an easily available and cost‐effective way of delivering care,^9^ we believe Tät® could be recommended to all women who want to perform PFMT via self‐management.

Future studies should aim to include more women that have had cesarean sections to be able to evaluate whether the mode of delivery could predict the response to PFMT. Further, it would be interesting to investigate how factors such as body mass index, comorbidity, and obstetric complications affect the results achieved by using the app.

## 5 CONCLUSION

A mobile app focused on PFMT is widely used by pregnant and postnatal women in Sweden both for preventing and treating UI. A majority of the pregnant and postnatal women with incontinence experienced improvement after 3 months of using the app Tät®. Postnatal incontinent women improved more in ICIQ‐UI SF score and had larger subjective improvements than pregnant incontinent women. User characteristics gave no support for only recommending the app to certain groups of pregnant and postnatal women. Regular PFMT and use of the app seemed to be important factors for experiencing improvement in incontinence symptoms.

## AUTHOR CONTRIBUTIONS

**Ina Asklund and Erika Löjdahl**: Conceptualization. **Erika Löjdahl and Anna Lindam**: Formal Analysis. **Ina Asklund**: Funding acquisition. **Erika Löjdahl, Ina Asklund, and Anna Lindam**: Writing–review and editing. **Erika Löjdahl**: Writing–original draft. All authors have read and approved the final version of the manuscript.

## CONFLICT OF INTEREST

The logos Tät and Tät.nu are registered as trademarks by The Swedish Patent and Registration office for eContinence AB, a Swedish e‐health company founded in July 2021, with the aim to maintain, spread, commercialize, and further develop the apps created within the research project Tät.nu (eContinence.se). Ina Asklund is a co‐founder and shareholder of eContinence AB. eContinence AB had no involvement in study design; collection, analysis, and interpretation of data; writing of the report; or the decision to submit the report for publication.

## ETHICS STATEMENT

The study was approved by the Regional Ethical Review Board, Umeå University (2014‐389‐32M, 2016‐80‐32M, and 2017‐405‐32M added to 2012‐325‐31M).

## TRANSPARENCY STATEMENT

Ina Asklund affirms that this manuscript is an honest, accurate, and transparent account of the study being reported; that no important aspects of the study have been omitted; and that any discrepancies from the study as planned (and, if relevant, registered) have been explained.

## Data Availability

The data that support the findings of this study are available from the corresponding author upon reasonable request. Erika Löjdahl had full access to all of the data in this study and takes complete responsibility for the integrity of the data and the accuracy of the data analysis.
